# Breast Developmental Anomalies in Dormaa Municipality of Ghana: Prevalence and Impact on the Life of the Individual

**DOI:** 10.1155/2013/140704

**Published:** 2013-03-19

**Authors:** P. Agbenorku, E. Otupiri, S. Fugar

**Affiliations:** ^1^Reconstructive Plastic Surgery and Burns Unit, Komfo Anokye Teaching Hospital, School of Medical Sciences, Kwame Nkrumah University of Science and Technology, Kumasi, Ghana; ^2^Department of Community Health, School of Medical Sciences, Kwame Nkrumah University of Science and Technology, Kumasi, Ghana; ^3^Department of Surgery, Komfo Anokye Teaching Hospital, Kumasi, Ghana

## Abstract

*Background*. Breast developmental anomalies (BDAs) are abnormalities of breast tissue that arise during breast development. Some of the anomalies can have negative impact on the person's life. This study seeks to assess the prevalence of BDA in the Dormaa Municipality in Ghana and its impact on the life of the individual. *Materials and Methods*. A descriptive study involving 500 female respondents aged between 11 and 25 years from selected schools in the Dormaa Municipality using self-administered questionnaires and interviews. *Results*. From the study, it was found that the prevalence of BDA in the municipality was 12.8%. The commonest BDA was bilateral hypoplasia which accounted for 31.3% of the BDAs found in the study. Nine (14.1%) complained of the BDA affecting their lives with most being teased in school. Twenty-two (34.4%) girls out of the 64 with BDAs had a family member with a BDA. *Conclusion*. BDA is a worry; therefore, comprehensive educational programs for health workers and the general public are needed to increase awareness. Also, work should be done to include education on BDA when awareness is being raised about breast cancer and on the importance of breast self-Examination (BSE).

## 1. Introduction

Since the beginning of time, the female breast has been a symbol of feminism, and its presence is a major feature that delineates a man from a woman. A beautiful and attractive female breast is one which is symmetrically situated on the anterolateral chest wall and has soft but well-defined junctures with the chest, upper abdomen, and the axillae. The breast profile is a gentle downward vertical flow from the clavicle extending between the second and sixth ribs, vertically and horizontally between the lateral edge of the sternum and midaxillary line to the nipple-areola and forms a mildly convex curve from the nipple-areola to the inframammary crease [1, 2].

Budding of the breasts (thelarche), a sign of female secondary sexual characteristic, occurs at approximately 10-11 years, and the time it takes for the breast to reach maturity can be as short as 18 months or may take as long as nine years. During this period of breast development, several processes or factors may go wrong leading to their abnormal growth. These include genetic, environmental, exposure to infectious agents, trauma, radiation, neoplastic, or endocrine conditions [3–7].

Some of these anomalies may arise purely in the breast tissue and these include amastia (absence of breast; occurs when mammary ridges fail to develop) [8], hypoplasia (underdeveloped breasts), macromastia (massive enlargement of one or both breasts; also defined as breast enlargement exceeding 600 grams) [9], and tuberous/tubular breast (breast with a tube-like shape; caused by an incomplete development of the mammary gland) [10]. Other breast anomalies are accessory nipples (polythelia) and accessory mammary glands (polymastia); these are believed to arise from an incomplete regression of the milk lines that arise embryologically [11].

Cases of BDAs have been recorded since the sixteen hundreds; one such case was described in 1670 where an autopsy performed on a patient who apparently died shortly after the onset of breast enlargement that weighed 64 pounds. Several other cases have been reported since then. One such case was described in 1993, in which a 12-year-old girl had abnormally large breast and developed marked kyphosis as a result. Apart from the physical impairment, the enlarged breast (hypertrophy) caused the girl intense psychological problems, incapacitating her in school activities and social relations [12]. Also, this form of large breasts has caused some people to stop engaging in normal daily activities because of the weight [13]. It has also been found that in the majority of these patients, they are young and healthy, but the psychological and social impacts of these conditions are crippling [14]. Other problems encountered by some women include physical disabilities such as constant back pain, scoliosis, as well as psychological problems as a result of insecurities about their beauty and feminism, and also teasing from peers [15].

Despite this long history of BDAs, there is a scarcity of research into these conditions. The importance of BDA has reached the point where centers are being set up in the US solely dedicated to management BDAs among adolescents [16]. Studies to investigate the awareness of breast anomalies such as performed by Agbenorku et al. in Jamasi revealed that the awareness of the breast anomaly was 83% among the people suffering from the BDA [17]. 

Diagnosis of BDAs is basically clinical and usually requires clinical examination of the breast. Careful breast examination is needed to prevent misdiagnosis because these anomalies may mimic breast malignances; so, it is important to rule them out [18]. Ultrasound is the ideal imaging modality in evaluating children's breasts [19]. Management of patients with BDAs requires a multidisciplinary approach which includes plastic surgeons, adolescent internists, endocrinologists, gynaecologists, psychiatrists, social workers and nutritionists. The whole team works primarily to fix any psychological problems that may exist first and secondly to correct the anomaly surgically if desired [20].

In patients with macromastia or gigantomastia, drugs are only marginally effective in reversing the condition; therefore, surgery (reduction mammaplasty) remains the mainstay of treatment [21]. When reduction mammaplasty is being considered in adolescent, surgery should be delayed until breast growth is completed, and this can be done by performing serial breast measurements [22]. Good skin care is also necessary in order to reduce breast crease inflammation and lessen the symptoms caused by moisture [23]. Tuberous breast can be treated by surgical procedures using tissue expansion methods [24]. Breast implants or reduction may be used to correct asymmetry.

Generally, the rate of patients who undergo surgery for BDA depends on the level of knowledge they have on the condition. Agbenorku et al. reported in their study that surgery and other forms of treatment such as counseling and regular followup assessment in patients with BDA were minimal, and in terms of treatment, 68% were ready to accept medication as the only treatment for their BDA [25].

### 1.1. Surgical Techniques for BDA Management

The most important aspects of the anatomy of the breast are the understanding of the blood supply and nerve supply to the nipple areola complex (NAC) [26]. It is also important to note that the inferior pedicle has been most associated with an inverted T- skin resection, a superior pedicle/supermedial pedicle with a vertical skin resection. The advent of pedicled techniques improved greatly NAC viability and improving cosmetics results [27]. Studies have shown that medial and superomedial pedicles have excellent postoperation nipple sensation [28]. Agbenorku et al. carried out bilateral reduction mammaplasty using the nipple-areola reposition technique which aims to preserve the nipples and some areolar tissue to retain sensibility and future breastfeeding. They reported in their study that reduction mammaplasty plays a significant role in the relief of pain and psychological distress of symptomatic macromastia patients, irrespective of available resources, technology, and country [25].

The technique employed is based on the individual patient's requirement. These include horizontal pedicle (which was the Strombeck pattern, with the blood supply coming from both sides) in which the dermal pedicle is sufficient to maintain nipple areolar viability but can cause nipple retraction and inclusion of glandular element in the pedicle which can make insetting difficult [29]. Inferior pedicle helps to maintain the NAC, and there is good circulation and sensation and breastfeeding possibility [30, 31]. Medial pedicle has good sensation and good blood supply and can be inserted relatively easy [32]. Lateral pedicle has good visibility and is based on the lateral thoracic artery perforators [33]. Superior pedicle has good circulation, but being a dermal pedicle, breastfeeding is impossible; it is not very easy to inset [34]. Central pedicle is a modification of the inferior pedicle with the removal of the dermal bridge [34]. Vertical bipedicle provided good blood supply and also easy inset [35].

### 1.2. Surgery and Psychology

The importance of psychological counseling of patients with BDA cannot be understated. It is important because in a study by Von Soest et al. amongst 130 Norwegian females who had cosmetic surgery, it was found that a high rate of preoperative psychological problems and low self-esteem were related to more negative psychosocial changes after the surgery as compared to those with better psychological health [36]. Thus, it is important to tackle the psychological aspect ideally even before the surgical correction takes place. Psychological management is also important because some of the individuals with these breast anomalies have an underlying body dysmorphic disorder [37].

## 2. Materials and Methods

### 2.1. Study Setting

Dormaa Municipal is located at the western part of the Brong-Ahafo Region of Ghana. It lies within longitudes 3° west and 3° 30′ west and latitudes 7° north and 7° 30′ north. Jaman and Berekum Districts, bound the district on the north, on the east by the Sunyani Municipal, in the south and southeast by Asunafo and Asutifi Districts respectively, in the southwest by Western Region, and in the west and northwest by Cote d'Ivoire. The municipal capital is Dormaa Ahenkro, located about 80 kilometers west of the regional capital, Sunyani [38]. The 2002 population census put Dormaa Municipality population at 150,229. It is estimated that Christians constitute the largest percentage of the district's population, accounting for 72% of the sampled population; Islam was 19%, while other religious groups represented 3%.

### 2.2. Materials and Methods

**Figure 1:**
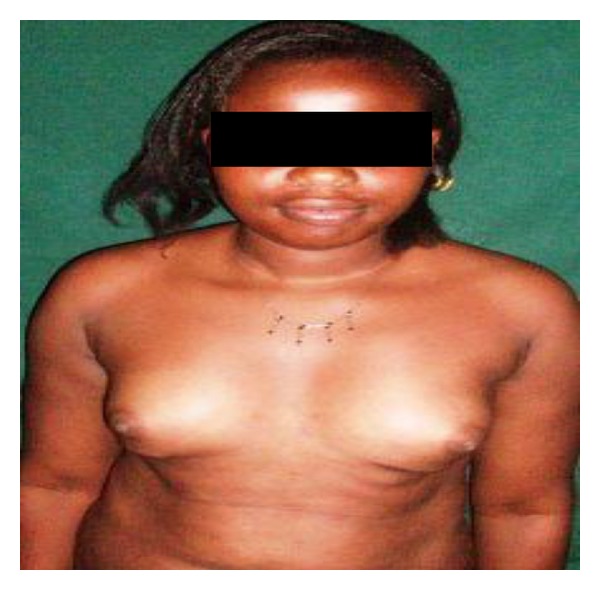
Bilateral hypoplasia in a 20-year-old.

**Figure 2:**
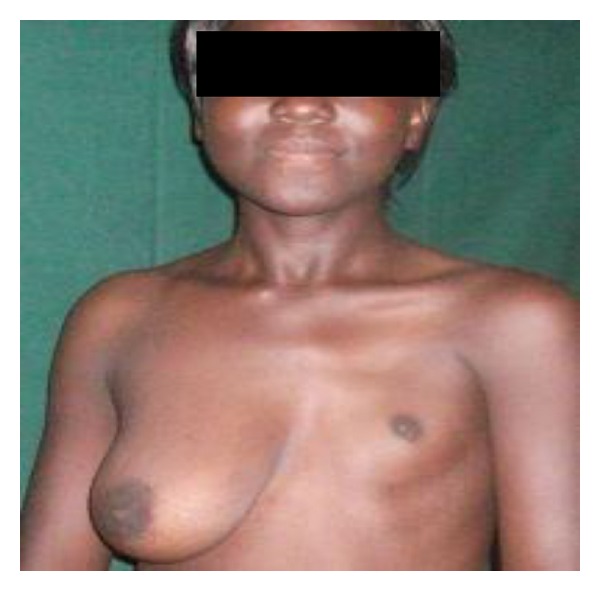
Left unilateral hypoplasia in a 22-year old.

**Figure 3:**
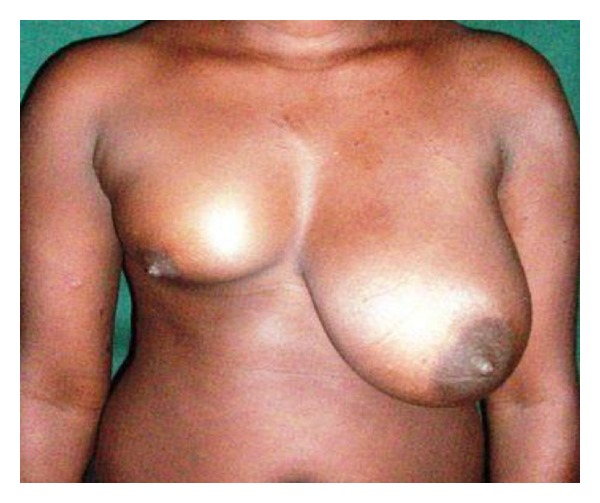
Left hyperplasia and right hypoplasia in a 16-year-old.

**Figure 4:**
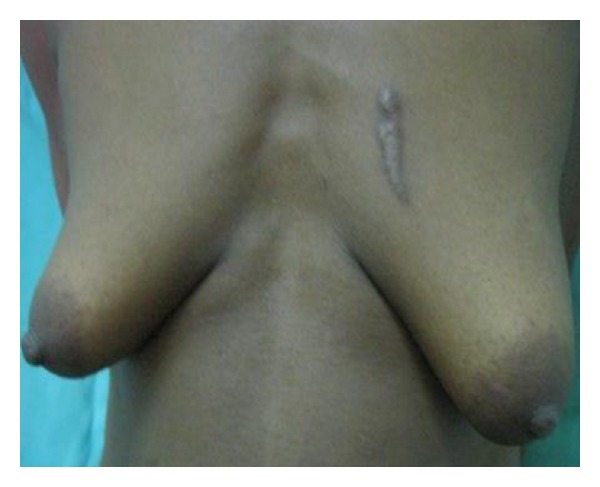
Bilateral tubular breasts in a 17-year-old.

**Figure 5:**
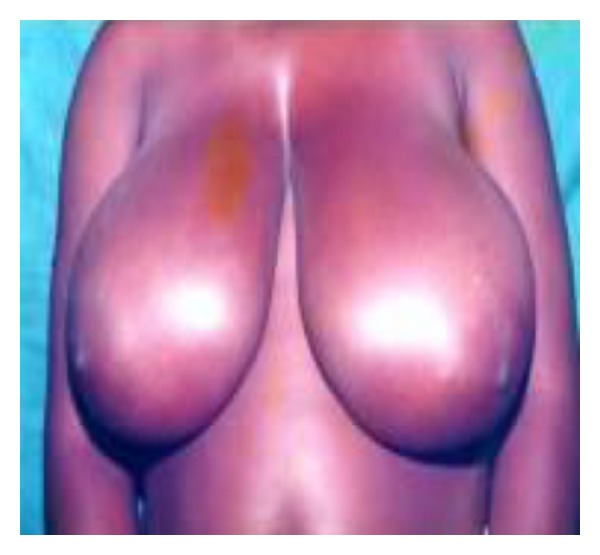
Bilateral juvenile macromastia in a 14-year-old.

**Figure 6:**
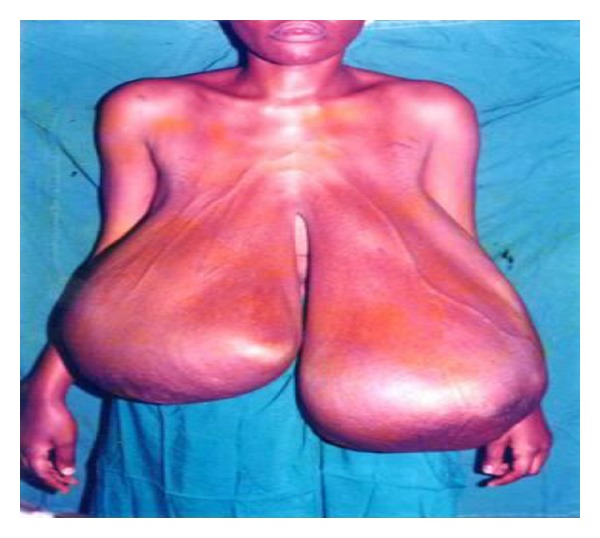
Bilateral breast hypertrophy in a 26-year-old.

**Figure 7:**
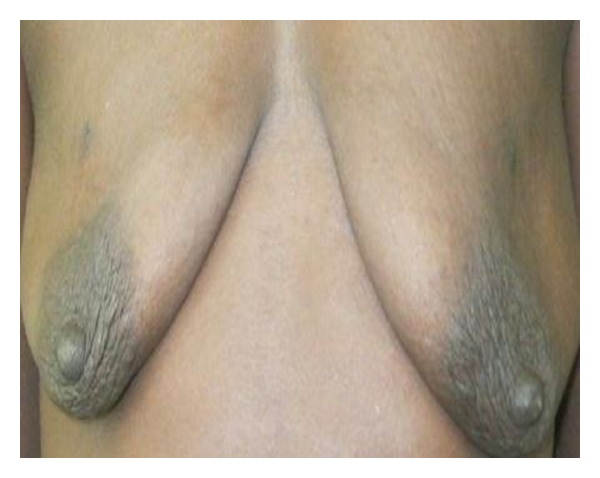
Bilateral accessory nipples in 45-year-old.

**Figure 8:**
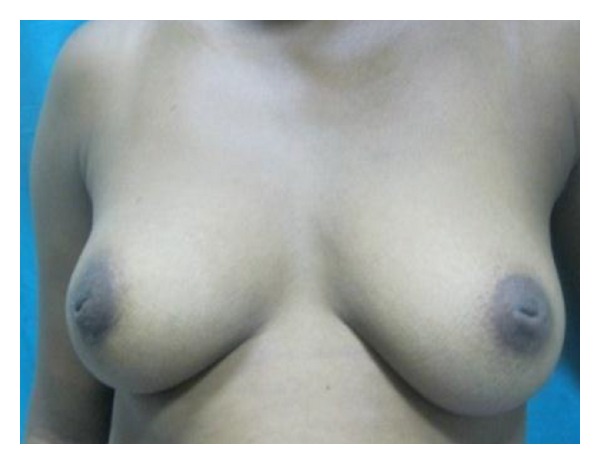
Bilateral nipple anomalies (Slit nipples) in a 23 year-old.

This descriptive study was conducted between February and April 2011. Consent was sought from the various leaders of the community and schools before the questionnaires were administered. The purpose of the study was explained to them after which the questionnaires were administered to a total of 500 hundred female students; 307 from senior high school (SHS) and 193 from junior high school (JHS) aged 11 to 25 in the municipality. The questionnaire used in the collection of data, developed from previous studies performed by Agbenorku et al. on BDAs at Jamasi and Sogakope, consisted of both open- and closed-ended questions [17, 28, 39]. Each respondent was shown 8 pictures of various BDAs (Figures 1, 2, 3, 4, 5, 6, 7, and 8). 

They were then asked to state if their breasts were like any of them. If their breasts were not like any of the ones described, they then moved to a section to assess their satisfaction with their breasts and knowledge of any disease of the breast. If the respondent said that her breast was like one of those pictures, then she had to answer questions as to the kind of impact the BDA had on her life. 

### 2.3. Data Analysis

Data from completed questionnaires were analyzed using data processing software, Statistical Package for Social Sciences (SPSS) 16th Edition. 

## 3. Results

**Figure 9:**
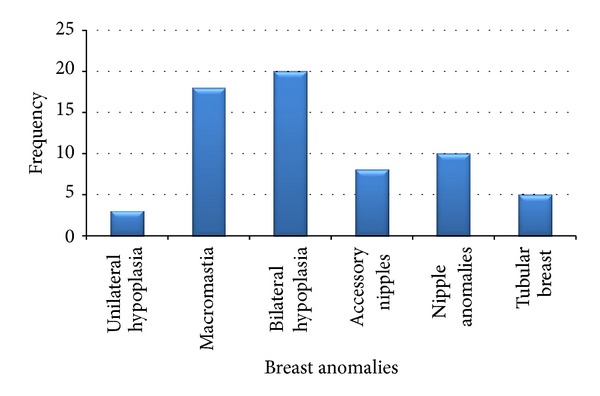
Forms of BDAs in respondents.

**Figure 10:**
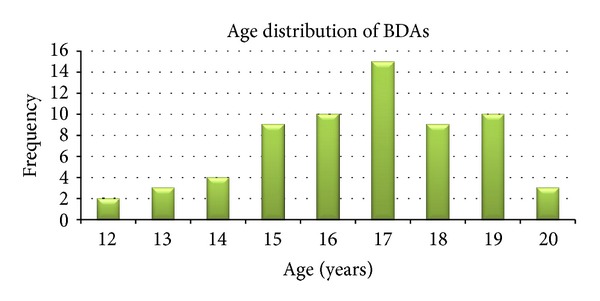
Age distribution of respondents with BDAs.

Figure 9 shows the distribution of the different anomalies. Out of the 500 respondents, 64 (12.8%) had BDA with the commonest being bilateral hypoplasia, 20 (31.3%), followed by macromastia 18 (28.1%).  Figure 10 illustrates the age distribution of female students with BDA. Most of the respondents (78.4%) were between the age of 15 and 20 years; 53 (82.8%) of those with BDA were between 15 and 19 years. The mean age of the girls with bilateral hypoplasia was 16.75 years with the youngest and the oldest being 14 and 20 years, respectively.

### 3.1. Problems Faced by Affected Individuals in School and at Home

Out of the 64 respondents with BDA, 9 (5 of them had macromastia, while 4 had bilateral hypoplasia) complained that BDA affected their lives. The girls with the bilateral hypoplasia complained of being teased in school; however, their lives at home and in town were not affected in anyway. Three (3) girls out of the 5 with macromastia complained of the BDA affecting their life at home; when they had to do household chores, they developed back pain, and 2 complained of being shy in school because of the size of their breasts. 

### 3.2. Reasons for Not Seeking Medical Treatment

None of the girls with a BDA had sought any treatment because 50 (78.1%) out of the 64 girls did not know that their conditions were considered abnormal. Three (3) girls with bilateral hypoplasia had researched into treatment options for people with small breasts but were discouraged by the high cost of surgery. Also, 10 were not aware of any treatment available (Table 1). 

### 3.3. Prevalence of BDA in the Family

Out of the 64 girls with BDA, 22 (34.4%) said they had a family member with a similar condition; 6 admitted to having seen the anomaly in their mothers, 22 in their sisters, 5 in their aunt, and 1 person admitted seeing it in her grandmother (Table 2).

### 3.4. Attitude and Response of the Family and Community

Out of the 64 respondents, 14 of them were aware they had a BDA. Six (9.4%) openly complained to their families about the condition, and the family's response to all these respondents was that their condition was normal. Eight (12.5%) respondents failed to tell anyone about their condition because they felt too shy to tell anyone in their family. 

### 3.5. Knowledge of Breast Diseases

Out of the 500 females interviewed, 134 (26.8%) girls were not satisfied with their breasts; 97 (72%) would be willing to have surgery performed to improve the look of their breasts. Thirty out of 97 wanted the size of their breasts to be increased, 65 wanted their breasts to be reduced, while the 2 wanted to fix the droopy nature of their breasts (augmentation). Thirty respondents out of the 64 (46.9%) with BDA were willing to have surgical correction of their anomaly. On knowledge of breast disease, 367 (73.4%) of the girls had heard of breast cancer; when asked whether they knew anyone apart of themselves or anyone in their family with BDA, 27 (5.4%) admitted knowing someone with a BDA.

## 4. Discussion

In this study, the prevalence of BDA was 12.8%. This is similar to findings done in other parts of the country where the prevalence recorded by Agbenorku et al. was 13% and 12.7% in Jamasi and Sogakope, respectively [17, 39]. More research on BDA has to be conducted, since prevalence in other areas could be high.

From this study, the commonest BDA found among the respondents was bilateral hypoplasia, followed by macromastia. Bilateral hypoplasia was reported much more frequently (30.7%) in this study than in other similar studies done in Ghana: 17.1% in Sogakope and 21.8% in Jamasi. In a study carried out by Simmons [16] among adolescents in the US, it revealed that polythelia and asymmetry were the most common BDAs. The prevalence of BDAs in this present study and other studies in Ghana was relatively higher than that found among Japanese where studies on young females revealed a prevalence of accessory nipples to be 5% [40, 41]. Some people may be of the view that breasts of the girls had not begun developing explaining the high prevalence of bilateral hypoplasia, but this cannot be the reason for the high rate of breast hypoplasia because the average age of the girls with bilateral hypoplasia was 16.75 years when in general most girls' breasts begin to develop between the ages of 10 and 11 years; therefore, the higher rate of breast hypoplasia may be attributed to other factors. One possible factor is that it could have arisen from the fact that this study design was a subjective design and unlike the other studies performed in the other areas where there was actual breast examination by the research team; so, the person's perception of their own breasts played a huge role in determining their choice.

Macromastia accounted for 28.1% of the BDAs. This value is not as high as those from other studies done in Ghana by Agbenorku et al. in Jamasi and Sogakope where macromastia accounted for 43.6% and 40.0%, respectively [17, 39].

A total of 34.4% of the respondents with a BDA had a family member with a similar condition (Table 2). This is similar to 31% reported by Agbenorku et al. in Jamasi [17]. This could mean that there is likely a genetic predisposition to the inheritance of BDA. Haagensen reported that there is a genetic element to BDAs when he noticed that the occurrence of polymastia or polythelia was inheritable condition [42]. 

In this study, 21.9% were aware that they had a BDA. This is quite low as compared to the awareness level of 63% recorded by Agbenorku et al. in Jamasi [17]. Awareness on BDA needs to be put in place in order to sensitize the general public. Regular breast self-examination (BSE) could result in early detection of these anomalies. A study conducted in Saudi Arabia revealed only 30.3% of the women had heard about BSE and 18.7% reported they practiced BSE within the previous year [43]. Also, a similar study undertaken in Nigeria by Okobia et al. showed that women lacked enough knowledge about breast cancer; 34.9% claimed to have ever-practiced BSE [44]. 

From the study, 67% wanted a reduction of their breasts. However, this is in contrast to a survey undertaken by Frederick on 26,703 American female adults where it was found that 70% of females were dissatisfied with their breasts and wanted more ample breasts [45]. This in response between the Ghanaians and Americans could be as a result of the fact that society plays a major role dictating the “ideal breast size,” and this perception would strongly influence their decision to either reduce or increase their breasts. In the United States, there is constant social and media pressure for women to have big breasts. 

Surgery is the best treatment option for BDA. As much as 46.9% respondents wanted to have surgical correction of their breasts. This rate is much higher than that seen in the study in Jamasi, where 20.5% of the girls with the BDA were willing to have surgical correction of their breasts [17]. The higher rates of acceptance of surgery in this study may arise from the fact that in this study the girls were not given any other treatment option such as drugs as a method of treatment which was an option in the Jamasi study. The treatment options were limited to surgical management because drug therapy is not effective. 

BDA could have such negative impacts on the lives of people. From this present study, 14.1% (*n* = 9) said that the anomaly was affecting their life in school or at home negatively. However, the situation in this present study differs from a similar study conducted by Agbenorku et al. at Jamasi which revealed that among those with BDA, it had no effect on their school and family life [17]. Benditte-Klepetko et al. carried out a study on 50 women with various breast sizes and concluded that that high breast weight has a negative influence on the physical and psychological morbidity of women [46]. Gözü et al. also stated in his study on a 12-year-old girl suffering from breast hypertrophy that although the deformity is benign, it affects patients physically and psychologically [47]. Somma et al. reported a case of a 12-year old girl with juvenile hypertrophy of breast which caused her intense psychological problems, incapacitating her in school activities and social relations [18]. More education is therefore needed to help persons with BDA to develop a positive self image.

## 5. Conclusion

The study revealed that the prevalence of breast developmental anomalies in the Dormaa Municipality is high. The study also showed that some girls are negatively affected by this condition; hence, education on BDA should be promoted to increase awareness of the condition. This form of education should also enlighten the public on treatment measures available. People should also be educated and encouraged to practice breast self-examination.

## Figures and Tables

**Table 1:** Reasons for not seeking treatment.

Reason	Frequency	Percentage
Not aware of disease	50	78.1
Not aware of treatment	10	15.6
Too expensive	3	4.7
Has no time for treatment	1	1.6
Any other reason	0	0

Total	64	100%

**Table 2:** Prevalence of BDA in the family.

BDA	Total cases seen among respondents	People with the same condition in their family	% with occurrence in the family
Unilateral hypoplasia	3	0	0%
Macromastia	18	5	27.8%
Bilateral hypoplasia	20	8	40%
Accessory nipples	8	3	37.5%
Nipple anomalies	10	4	40.0%
Tubular breasts	5	2	20%

Totals	64	22	34.4%
